# Montelukast reduced docetaxel-induced peripheral neuropathy in rats by altering oxidative stress, histopathological damage, and gene expressions

**DOI:** 10.1590/1414-431X2025e14602

**Published:** 2025-05-30

**Authors:** M.D. Karakoç, Ö. Özmen, M.N. Zengin, O. Çiftçi

**Affiliations:** 1 Department of Medical Pharmacology, Faculty of Medicine, Pamukkale University, Denizli, Turkey; 2 Department of Pathology, Faculty of Veterinary Medicine, Burdur Mehmet Akif Ersoy University, Burdur, Turkey

**Keywords:** Docetaxel, Montelukast, Antioxidant, Peripheral neuropathy, Neurotoxicity

## Abstract

Peripheral neuropathy (PN) is a common side effect of docetaxel (DTX). In this study, we aimed to evaluate the effects of montelukast (MNT), a leukotriene receptor antagonist drug, against DTX-induced PN in rats. Thirty-two male rats were divided into four groups and treated for four weeks: control (sham), DTX (5 mg/kg per week, *ip*), MNT (10 mg/kg per day, *po*), and DTX+MNT (5 mg/kg per week, *ip* + 10 mg/kg per day, *po*). Behavioral tests (hot plate, tail flick, and rotarod) were conducted. Histopathological, molecular (RT-PCR), and biochemical (ELISA) analyses were performed on sciatic nerve, liver, and serum samples. MNT reduced the malondialdehyde (MDA) levels and increased the superoxide dismutase (SOD), catalase (CAT), and glutathione (GSH) parameters in sciatic nerve tissues. Unlike DTX, MNT resulted in increased *Bcl-2* gene expression and decreased caspase-3 (*Cas-3*) and *Bax* expressions. DTX caused sensory and motor neuropathy, as revealed by the hot plate, tail flick, and rotarod tests. The co-administration of MNT significantly mitigated the sensory and motor neuropathy induced by DTX. MNT improved the levels of NCAM, p38α MAPK, and nuclear factor kappa B (NF-κB), which were impaired in the sciatic nerve tissues due to DTX administration. Additionally, it reduced the levels of tumor necrosis factor-α (TNF-α) and interleukin-6 (IL-6), which had increased due to DTX. Histopathological examination revealed that DTX-related sciatic nerve damage was mitigated by MNT administration. The results indicated that MNT may have a protective effect against DTX-induced PN in rats.

## Introduction

Peripheral neuropathy (PN) is a significant adverse effect of cancer chemotherapy. It affects approximately 48% of patients undergoing treatment, with 68% developing chemotherapy-induced PN within the first month of therapy. Chemotherapy-induced PN is primarily characterized by sensory axonal neuropathy, manifesting as numbness and paresthesia in the extremities. Many patients also experience fatigue and severe pain (1). Proposed mechanisms for chemotherapy-induced PN include DNA damage, excessive oxidative stress, increased inflammation, mitochondrial dysfunction, microtubule disruption, impaired ion channel activity, and myelin sheath damage, all triggered by antineoplastic drugs (2). Up to 40% of patients with chemotherapy-induced PN continue to experience symptoms such as pain and numbness that significantly impair their quality of life even after they complete their treatment (3).

Various antineoplastic agents are associated with neurotoxicity and chemotherapy-induced PN, with docetaxel (DTX) standing out as one of the most likely to cause these side effects (4). DTX, a widely used semi-synthetic taxane derivative, is employed either alone or in combination with other drugs to treat various solid tumors. It exerts its effects by inhibiting the disassembly of tubulin from the microtubule polymer (5). Severe PN symptoms are frequently observed with DTX administration, which not only diminishes patients' quality of life but can also lead to treatment delays, dose reductions, the use of less effective alternatives, or even the discontinuation of therapy, ultimately resulting in suboptimal treatment outcomes (6). To date, numerous preclinical and clinical studies have been conducted to prevent DTX-induced PN. Proposed interventions include physical therapies such as exercise, cryotherapy, and acupuncture as well as pharmacological agents such as amifostine, N-acetylcysteine, nimodipine, acetyl-L-carnitine, and vitamin E. Additionally, chemical compounds such as glutathione, omega-3 fatty acids, all-trans retinoic acid, calcium, and magnesium have been tested. Unfortunately, no effective compounds or protocols have yet been identified to prevent this serious chemotherapy-related toxicity (7).

Montelukast (MNT) is a cysteinyl leukotriene receptor antagonist. It acts as a selective antagonist, primarily against leukotriene D4, inhibiting its effects. Leukotriene receptor antagonists such as MNT are commonly used to treat respiratory diseases, particularly asthma and allergic rhinitis (8,9). In recent years, numerous studies have demonstrated that MNT exhibits significant antiapoptotic, neuroprotective, and antioxidant properties in addition to its established anti-inflammatory effects (10-12). These additional pharmacological activities suggest that MNT may help mitigate DTX-induced neurotoxicity. Therefore, we aimed to investigate the effects of MNT on DTX-induced PN in rats by examining behavioral and histopathological changes as well as assessing apoptosis-related gene expressions and biochemical alterations in serum and sciatic nerve samples. Furthermore, we explored whether the two drugs influence each other's metabolism via CYP450 enzymes through biochemical analyses of liver samples.

## Material and Methods

### Chemicals

MNT (Onceair^®^) was provided by Abdi İbrahim İlaç San. A.Ş. (Turkey). DTX (Doxel Ready^®^) was obtained from Gensenta İlaç San. A.Ş. (Turkey). All other chemicals were purchased from Sigma-Aldrich Chemical Co. (USA) and were of analytical grade or the highest grade available.

### Animals and ethics

The experimental protocol was approved by the Local Ethics Committee for Experiments on Animals of Pamukkale University (protocol No. PAUHDEK-2023/09). Thirty-two healthy adult male Sprague-Dawley rats (3 months old and 215-245 g in weight) were obtained from Pamukkale University Experimental Surgery Application and Research Center (Turkey) for the experiment. The animals were housed in polypropylene rat cages and underwent a 12-h light/dark cycle at an ambient temperature of 23±2°C and relative humidity of 55-60%. Standard rat chow and water were provided *ad libitum*. The rats were given one week to acclimate to the laboratory conditions. Animal care and experimental procedures were carried out according to the National Institutes of Health Guidelines for the Use of Laboratory Animals.

### Experimental design and drug treatment

The rats were randomly divided into four groups, with eight animals in each group: control, DTX, MNT, and DTX+MNT. The body weight of the rats was monitored daily. MNT was suspended in distilled water (DW) before each oral administration. The control group rats were administered 0.2 mL of 0.9% saline solution intraperitoneally (*ip*) once a week, and they received 1 mL of DW by oral gavage (*po*) per day for 4 weeks (approximately equal drug volumes to those in other groups). The DTX group animals were administered 5 mg/kg of DTX (*ip*) once a week and 1 mL of DW (*po*) per day for 4 weeks. The rats in the MNT group were administered 10 mg/kg of MNT (*po*) per day and 0.2 mL of 0.9% saline solution (*ip*) once a week for 4 weeks. The rats in the DTX+MNT group were administered 5 mg/kg DTX (*ip*) once a week and 10 mg/kg MNT (*po*) per day for 4 weeks. The drug doses, routes of administration, and frequency of application in this study were determined based on previous research that reported the peripheral neurotoxicity of DTX (13) and the pharmacological benefits of MNT (14) in rats. The rats were euthanized 3 days after the last administration under anesthesia (50 mg/kg ketamine, *ip*, and 5 mg/kg xylazine, *ip*). Blood samples were taken by cardiac puncture, and serum was separated for biochemical analysis. Livers and sciatic nerves were removed immediately. Liver tissues were solely utilized for determining cytochrome P450 (CYP450) enzyme levels by enzyme-linked immunosorbent assay (ELISA). Sciatic nerve tissues were used for ELISA, real-time polymerase chain reaction (RT-PCR), and histological and immunohistochemical analyses. Routine procedures detailed in the relevant section were followed for the histological analysis of right sciatic nerve tissues. The remaining tissues were stored at −80°C until analysis.

### Behavioral tests

The hot plate and tail flick tests are commonly used methods to assess pain sensitivity caused by thermal hyperalgesia in rats. Additionally, the rotarod test is frequently employed to evaluate motor performance and balance in rats on a rotating rod. All behavioral tests were conducted on the same days, with the familiarization for the hot plate and tail flick tests coinciding with the final pre-training day of the rotarod test. Each of the tests was performed on all the animals in all the groups one day before the first drug administration (DTX) and one day after the last drug administration (final dose of MNT).

### Rotarod test

The rats were pre-trained on the rotarod (May^®^ RR 0711, Turkey) with four trials per day for three consecutive days at a constant speed of 10 laps per min. The initial and final tests were then performed at the same speed.

### Hot plate test

The rats were acclimated to the hot plate apparatus one day prior to the initial testing. Each animal was individually placed onto a hot plate (May^®^ AHP 0603) heated to 52°C. The time interval from the moment the animals' paws made contact with the plate to their initial paw withdrawal response was recorded.

### Tail flick test

Thermal hyperalgesia was measured using a May^®^ AHP 0703 tail flick analgesia meter. The animals were acclimated to the tail flick device one day before the initial testing. A pull or curl of the tail in response to pain indicates that the animal is feeling extreme pain. The cut-off time was set as 20 s.

### Preparation of tissue homogenates

Liver and left sciatic nerve tissue samples were homogenized in a 10% 150 mM phosphate buffer solution (pH 7.4) using a Teflon-glass homogenizer at 2000 *g* for 1 min. Tissue homogenates were then centrifuged at 12,000 *g* at 4°C for 10 min. After centrifugation, supernatants were collected. Each supernatant was analyzed separately on the same day.

### Gene expression analyses by RT-PCR

The RT-PCR analyses were performed in accordance with MIQE guidelines (15). Messenger RNA (mRNA) expression analyses were performed using StepOnePlus^©^ RT-PCR (Applied Biosystems, USA) according to the A.B.T. 2X qPCR MasterMix kit (A.B.T. Laboratory Industry, Turkey) protocol. The RT-PCR conditions for the genes were as follows: one cycle of initial denaturation at 95°C for 10 min followed by 40 cycles of denaturation at 95°C for 30 s, and 40 cycles of annealing at 60°C for 60 s. β-actin was used as a reference housekeeping gene in the analysis of *Bcl-2*, *Bcl-2* associated × protein (*Bax*), and caspase-3 (*Cas-3*) mRNA expressions. The sequences of *Bcl-2*, *Bax*, *Cas-3*, and *β-actin* primers used for RT-PCR are presented in Table 1. The experiments were conducted in triplicate. In the analysis of the data, quantitation was performed using the ΔΔCT method in the “RT2 Profiler PCR Array Data Analysis” program on the internet-based Gene Globe platform (https://geneglobe.qiagen.com/us/analyze).

**Table 1:** Primer sequences used for RT-PCR.

Gene	Primers	NCBI Reference Sequence
*β-actin*	Forward: 5′-GACGATATCGCTGCGCTCG-3′ Reverse: 5′-CAATGCCGTGTTCAATGGGG-3′	NM_031144.3
*Bcl-2*	Forward: 5′-CATGTGTGTGGAGAGCGTCA-3′ Reverse: 5′-ACTCAGTCATCCACAGAGCG -3′	NM_016993.2
*Bax*	Forward: 5′-CAACATGGAGCTGCAGAGGA-3′ Reverse: 5′-GGAAAGGAGGCCATCCCAG-3′	NM_017059.2
*Cas-3*	Forward: 5′-CTTTGCGCCATGCTGAAACT-3′ Reverse: 5′-CAAATTCCGTGGCCACCTTC-3′	NM_001436900.1

*Bcl-2*: B-cell lymphoma 2; *Bax*: Bcl 2-associated × protein; *Cas-3*: caspase-3; NCBI: National Center for Biotechnology Information.

### Biochemical analysis

The hepatic metabolism of DTX and MNT in rats is mediated by CYP3A1 and CYP2C22, which are considered the rat orthologs of human CYP3A4 and CYP2C8, respectively (16-18). Therefore, we determined the expression levels of rat CYP3A1 using the BT LAB^®^ (China) ELISA kit (cat No. E1828Ra) and CYP2C22 using the ELK Biotechnology^®^ (USA) ELISA kit (cat No. ELK8937-Ra), following the manufacturers' instructions, in the liver tissues. Tumor necrosis factor-α (TNF-α) and interleukin-6 (IL-6) levels in the serum were determined using commercial Invitrogen^®^ (USA) rat ELISA kits (cat No. BMS622TEN and BMS625TEN, respectively), according to the manufacturer's instructions.

Supernatants from sciatic nerve tissues were used to assess the levels of specific parameters associated with PN, including nuclear factor kappa B (NF-κB), p38α mitogen-activated protein kinase (p38α MAPK), and neural cell adhesion molecule (NCAM), using ELISA. Analyses for NF-κB and p38α MAPK were conducted using Invitrogen^®^ commercial kits (cat No. EEL138 and 85-86022, respectively), while analysis for NCAM was performed using an ELK Biotechnology^®^ (USA) ELISA kit (cat No. ELK0369), following the respective manufacturers' instructions.

Antioxidant/oxidant status in sciatic nerve tissues was detected using a Shimadzu^®^ UV-1601 spectrophotometer (Japan). The antioxidant parameters evaluated were superoxide dismutase (SOD), catalase (CAT), and glutathione (GSH), while malondialdehyde (MDA) was examined as the oxidant parameter. SOD activity in tissues was measured at 560 nm according to a previously described method (19). The process is based on inhibiting nitro blue tetrazolium reduction using the xanthine oxidase system as a superoxide generator, with the results expressed as U/mg protein. The CAT activity was detected by measuring the disintegration of hydrogen peroxide (H_2_O_2_) at 240 nm, reported as k/mg tissue protein (20). The analysis of GSH in tissues was conducted following the procedure outlined by Tietze at 412 nm, with outcomes represented as mg/g protein (21). MDA analysis is based on the optical density at 532 nm of the color produced by MDA in thiobarbituric acid, according to the methodology described by Ohkawa et al. (22), and the findings are reported in nmol/mg protein. The total protein concentrations of the supernatants used to calculate the antioxidant/oxidant parameters were determined using commercially available kits employing the biuret method (Archem Diagnostic Ltd, Turkey).

### Histopathological analysis

During necropsy, the sciatic nerve was carefully excised, grasped at the tip with forceps, and immersed in formaldehyde solution. After removal, the nerve was placed on a wooden board and left for 5 min to harden. It was then transferred into tissue processing cassettes and re-immersed in formaldehyde solution. The tissue samples were processed using a fully automated tissue processor (Leica ASP300S; Leica Microsystem, Germany) and embedded in paraffin wax. Sections of 5-µm thickness were cut from the paraffin blocks using a fully automated Leica RM 2155 rotary microtome (Leica Microsystem). The sections were subsequently stained with hematoxylin-eosin (HE), coverslipped, and examined under a light microscope (Olympus CX41, Olympus, Japan). Histopathological findings were assessed based on the degree of edema, axonal vacuolization, and inflammatory cell infiltration. Each section was semi-quantitatively scored on a scale from 0 to 3 as follows: normal (0), mild (1), moderate (2), and severe (3).

### Immunohistochemical analysis

Additionally, three series of sections from each paraffin block were placed on poly-L-lysine coated slides and stained immunohistochemically for the expression of *Cas-3* (recombinant anti-caspase-3 antibody [EPR18297] (ab184787)), *Bcl-2* (anti-Bcl-2 antibody (ab196495)), and *Bax* (recombinant anti-*Bax* antibody [E63] (ab32503)) using the streptavidin-biotin peroxidase method according to the manufacturer's instructions. Each primary antibody was diluted to 1:100. The sections were incubated with the primary antibodies overnight followed by immunohistochemistry using a streptavidin-alkaline phosphatase conjugate and a biotinylated secondary antibody. The Mouse and Rabbit Specific HRP/DAB (ABC) Detection IHC kit (ab64264) was used as the secondary antibody, and diaminobenzidine (DAB) was employed as the chromogen. All primary and secondary antibodies were obtained from Abcam (UK). For negative controls, antigen dilution solution was used in place of the primary antibodies. Histopathological and immunohistochemical analyses were performed in a blinded manner by an expert histopathologist who is part of the research team and affiliated with another university.

The percentage of cells that were positively immunostained for each marker in 10 distinct fields for each slide for all groups was calculated at an objective magnification of ×40. Counting was performed on the image analyzer's output using the ImageJ program (National Institutes of Health, USA, version 1.48). The photos were separated into color channels and cropped, and any artifacts were eliminated before counting. Cells within the regions of interest were counted using the software's counting tool after being picked by a selection tool. Only cells that displayed intense brown staining were regarded as positive, and the brown hue was employed to determine positive staining. Morphometric analyses were performed using the Olympus CX41 light microscope and Axios camera. Microphotographs were taken using the Database Manual Cell Sens Life Science Imaging Software System (Olympus Co., Japan).

### Statistical analysis

Statistical analysis was performed using SPSS 29.0 (SPSS Inc., IBM, USA). Data are reported as means±SE. The suitability of the data for normal distribution and homogeneity of variance was determined using the Shapiro-Wilk and Levene's tests, respectively. Histopathological scores were analyzed using the non-parametric Kruskal-Wallis test, while differences between groups were assessed using a one-way analysis of variance (one-way ANOVA) with Dunnett's C test. For parametric values, we used a one-way ANOVA to evaluate differences between groups. To check the significance of differences, we used the *post hoc* Tukey test. Differences between initial and final measurements for certain parameters, such as rotarod, hot plate, tail flick, and body weights, for each group were analyzed using a paired samples *t*-test. In the RT-PCR analysis, quantitation was performed using the ΔΔCT method via the Gene Globe RT2 Profiler™ PCR Array Data Analysis online platform. The significance threshold was set at P<0.05.

## Results

### Behavioral test results

In the initial analyses conducted on all behavioral tests (rotarod, hot plate, and tail flick), no significant differences were found between the groups (P>0.05). On the other hand, when the results of the final tests were compared with the initial tests, significant differences were found for all parameters (P<0.001 for the rotarod and hot plate, P<0.011 for the tail flick test).

#### Rotarod test

**Figure 1:**
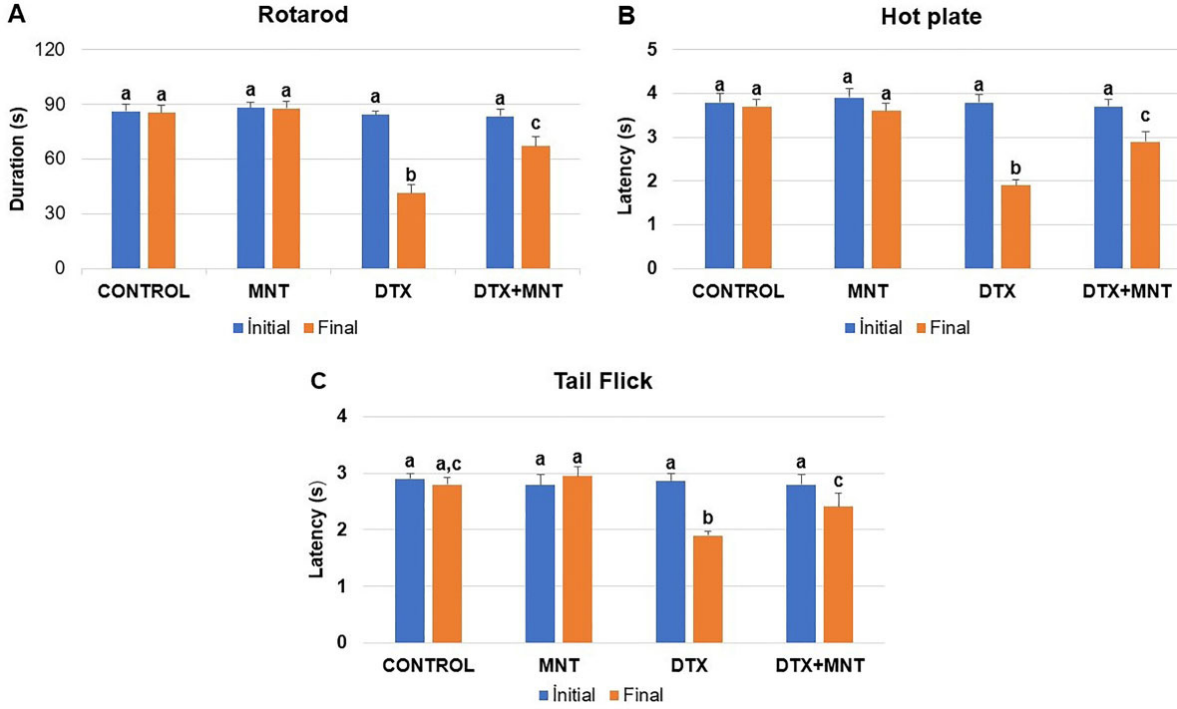
Results of the behavioral tests. Initial and final rotarod (**A**), hot plate (**B**), and tail flick (**C**) test results of rats. Results are reported as means±SE. Different letters (a, b, c) on the columns indicate a statistically significant difference with each other (P<0.05; Student’s *t*-test). MNT: montelukast; DTX: docetaxel.

The administration of DTX led to impairment in the motor coordination of rats (P<0.001). However, MNT attenuated the severity of the motor dysfunction caused by DTX (P<0.002) (Figure 1A).

#### Hot plate test

The paw withdrawal threshold was significantly reduced after DTX administration (P<0.001). On the other hand, oral administration of MNT significantly increased the threshold compared to the DTX‐treated group (P<0.002) (Figure 1B).

#### Tail flick test

DTX significantly increased the sensitivity to thermal hyperalgesia in rats (P=0.001). However, oral MNT use was found to considerably alleviate the toxicity caused by DTX (P<0.05) (Figure 1C).

### Gene expressions

**Figure 2:**
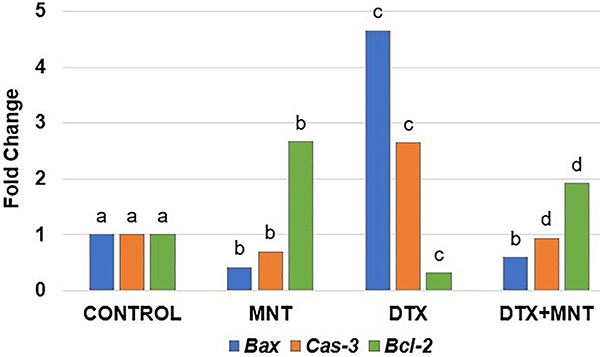
Fold changes of gene expressions in the sciatic nerve tissues of experimental groups relative to the control group. Different letters (a, b, c, d) on the columns indicate a statistically significant difference with each other (P<0.05; ANOVA). *Bcl-2*: B-cell lymphoma 2; *Bax*: Bcl 2-associated × protein; *Cas-3*: caspase-3; MNT: montelukast; DTX: docetaxel.

DTX led to a decrease in the expression of *Bcl-2*, an anti-apoptotic gene, while causing an increase in the expression of the apoptotic genes *Bax* and *Cas-3* in the sciatic nerve tissues. It was determined that the application of MNT resulted in a reduction in the expression of apoptotic genes such as *Cas-3* and *Bax* accompanied by an increase in the expression of the anti-apoptotic gene *Bcl-2*. The fold changes of the genes in the sciatic nerve tissues of experimental groups are presented in Figure 2.

### Biochemical analysis

**Figure 3:**
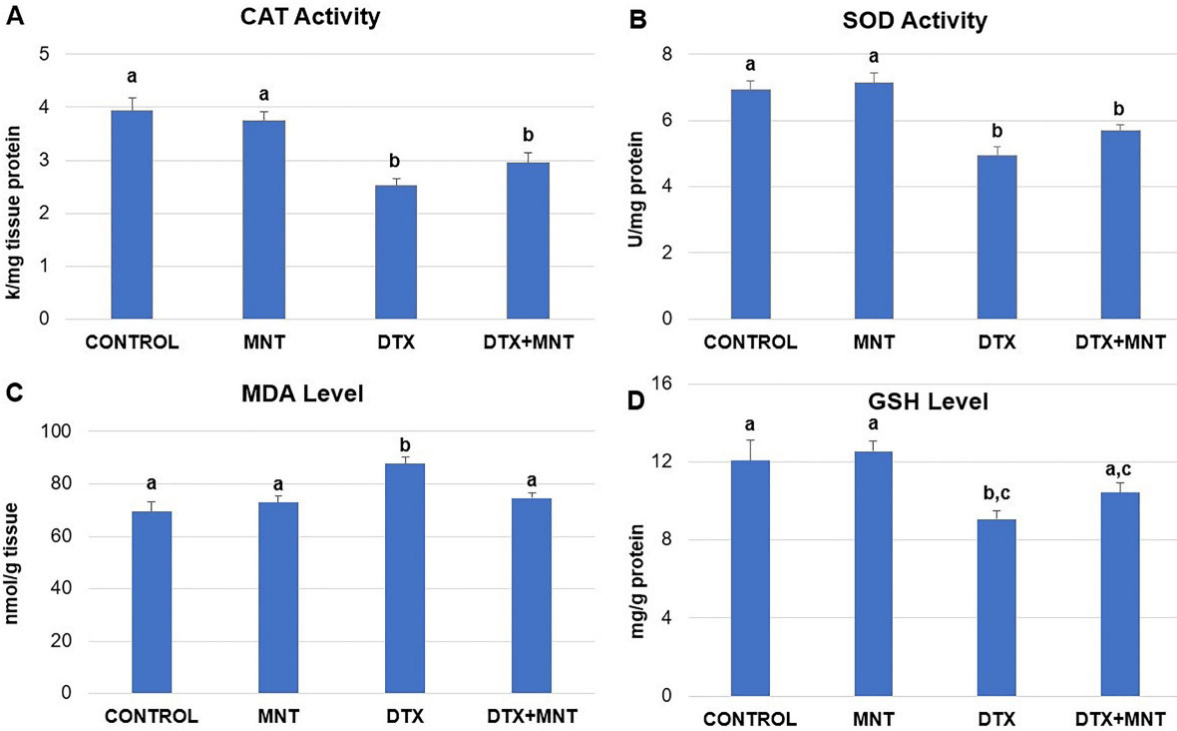
**A**, Distribution of catalase (CAT) activity, **B**, superoxide dismutase (SOD) activity, **C**, malondialdehyde (MDA) level, and **D**, glutathione (GSH) level in sciatic nerve tissues of experimental groups. Results are reported as means±SE. Different letters (a, b, c) in the columns indicate a statistically significant difference with each other (P<0.05; ANOVA). MNT: montelukast; DTX: docetaxel.

Administration of DTX led to a significant (P<0.001) decrease in the activities of CAT and SOD in the sciatic nerve tissues compared to the control group. Co-treatment with MNT (10 mg/kg body weight) resulted in a minor increase in CAT and SOD activities compared to the DTX-treated group; however, the differences were not statistically significant (P>0.05). Details of the CAT and SOD activities are presented in Figure 3A and B, respectively.

DTX administration significantly increased the level of MDA in sciatic nerve tissues compared to the control group (P<0.001). The MNT treatment significantly decreased the MDA level compared to the group treated with DTX only (P=0.012), bringing it to levels similar to those of the control group (P>0.05), as shown in Figure 3C.

DTX caused a notable decrease in GSH levels in sciatic nerve tissues (P<0.05). MNT co-administration resulted in a slight increase in GSH levels, elevating them to levels similar to those of the control group (P>0.05), as depicted in Figure 3D.

**Figure 4:**
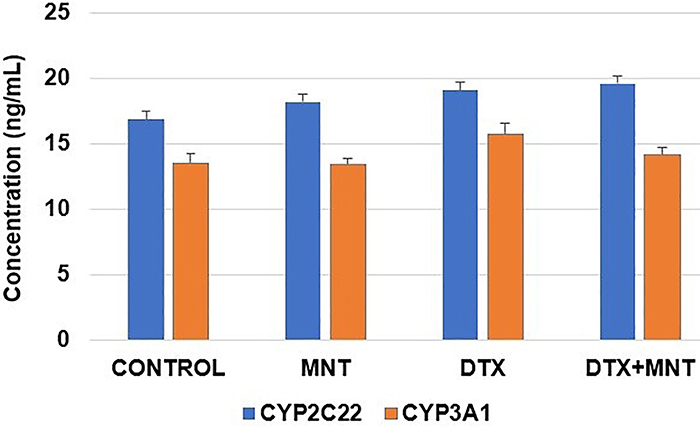
Levels of CYP3A1 and CYP2C22 enzymes in the liver tissues of the experimental groups. No significant differences were found between groups (P>0.05; *t*-test). Results are reported as means±SE. DTX: docetaxel; MNT: montelukast.

At the end of the one-month drug administration process, there were no significant differences between the experimental groups in terms of both CYP3A1 and CYP2C22 enzyme levels in liver tissues (P>0.05) (Figure 4).

**Figure 5:**
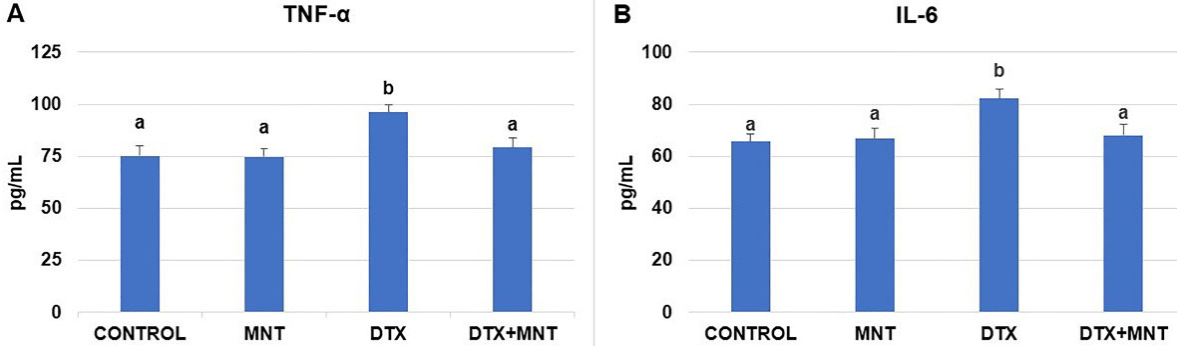
**A**, Distribution serum tumor necrosis factor-α (TNF-α) level and **B**, serum interleukin (IL)-6 level of experimental groups. Results are reported as means±SE. Different letters (a, b) in the columns indicate a statistically significant difference with each other (P<0.05; ANOVA). MNT: montelukast; DTX: docetaxel.

The administration of DTX resulted in a significant elevation in serum TNF-α and IL-6 levels compared to the control group (P<0.05 for both parameters). Conversely, MNT administration led to a significant decrease in the examined serum cytokine levels in DTX-treated rats. The difference between the DTX+MNT and the DTX groups was statistically significant (P<0.05 for each parameter). Furthermore, no significant differences were observed in TNF-α and IL-6 levels between the control, MNT, and DTX+MNT groups (P>0.05) (Figure 5A and B).

**Figure 6:**
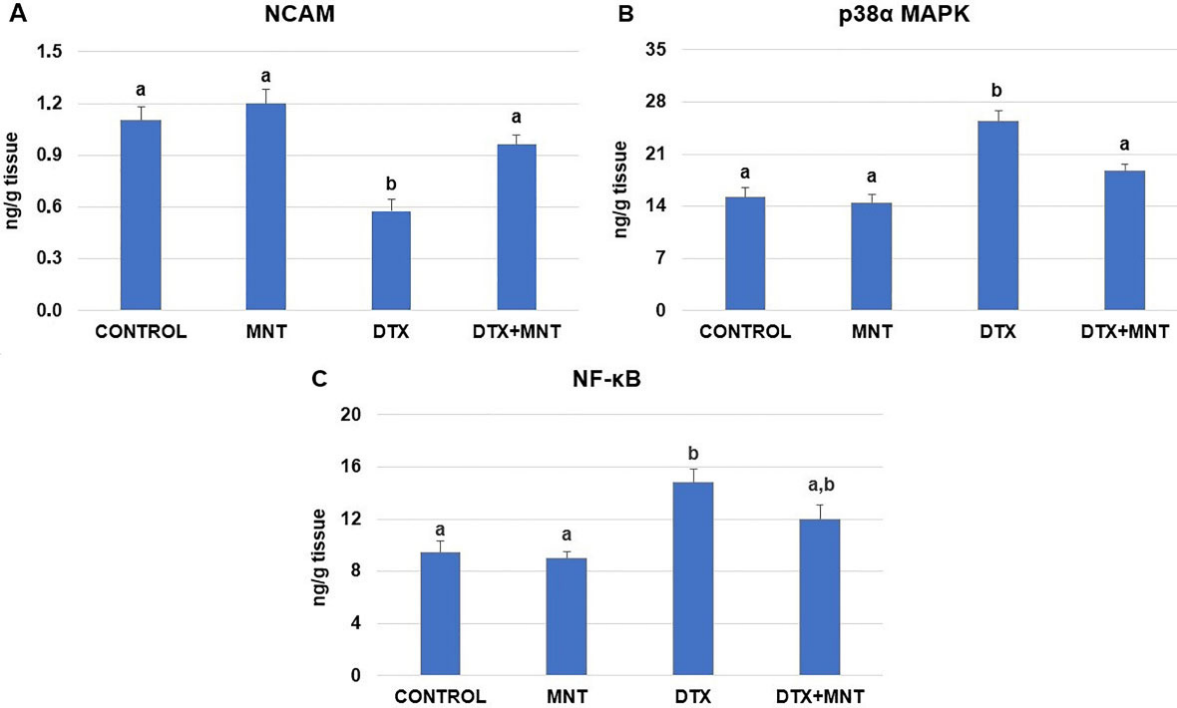
**A**, Effect of MNT on DTX-induced sciatic nerve NCAM levels, **B**, p38α MAPK activity, and **C**, NF-κB levels in rats. Results are reported as means±SE. Different letters (a, b) in the columns indicate a statistically significant difference with each other (P<0.05; ANOVA). MNT: montelukast; DTX: docetaxel; NCAM: neural cell adhesion molecule; p38α MAPK: p38α mitogen-activated protein kinase; NF-κB: nuclear factor kappa B.

Exposure to DTX resulted in a decrease in NCAM levels compared to the healthy control group (P<0.001), as shown in Figure 6A. However, treatment with 10 mg/kg of MNT throughout the chemotherapy cycles led to a significant increase in NCAM levels compared to the group treated with DTX alone (P=0.005). Furthermore, no significant differences in NCAM levels were observed among the control, MNT, and DTX+MNT groups (P>0.05).

To further investigate the anti-inflammatory effects of MNT in relation to DTX-induced sciatic nerve injury, we analyzed the effects of MNT treatment on the levels of NF-κB and p38α MAPK. DTX caused a significant (P<0.0001) increase in the level of p38α MAPK compared to the control and MNT-alone treated groups, as shown in Figure 6B. In contrast, the co-administration of MNT with DTX significantly decreased the level of p38α MAPK (P=0.003). No significant differences were observed in the levels of p38α MAPK among the control, MNT, and DTX+MNT groups (P>0.05). Similarly, DTX caused a significant increase in the level of NF-κB compared to both the control and MNT-alone treated groups (P=0.002 and P=0.001, respectively). The co-administration of MNT to DTX-treated rats resulted in a slight decrease in this parameter; however, the difference was not statistically significant between the DTX-alone and DTX+MNT groups (P>0.05). The distribution of NF-κB levels among experimental groups is presented in Figure 6C.

### Histopathological and immunohistochemical findings

**Figure 7:**
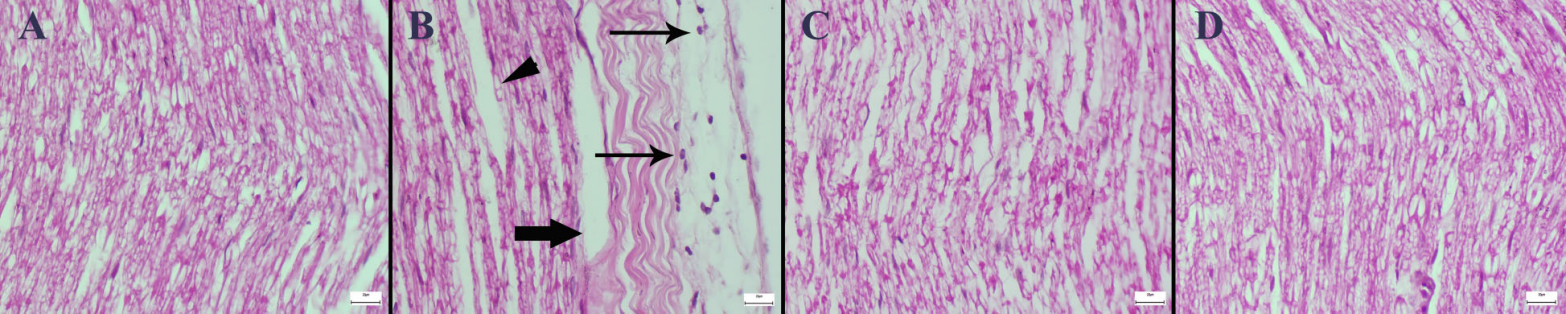
Histopathological appearance of sciatic nerve sections of the groups (hematoxylin-eosin). **A**, Normal tissue architecture in the Control group. **B**, Marked inflammatory cell infiltrations (thin arrows), axonal cytoplasmic vacuolization (arrowhead), and intramyelinic edema (thick arrow) in the DTX group. **C**, Decreased pathological findings in the DTX+MNT group. **D**, Normal nerve histology in MNT group. Scale bars=20 µm. MNT: montelukast; DTX: docetaxel.

The results of the histopathological examinations showed that DTX caused marked histopathological sciatic nerve damage characterized by inflammatory cell infiltrations, axonal cytoplasmic vacuolization, degeneration, and intramyelinic edema. In addition, MNT significantly reduced the histological findings in the DTX+MNT group. No pathological findings were observed in the control and MNT groups (Figure 7). The scoring criteria according to the IHAND terminology (23) and the number of animals per group are presented in Table 2. The statistical analysis results of the histopathological scores among the groups are shown in Table 3.

**Table 2:** Histopathological scores and the number of animals per group.

	Score	Control	DTX	DTX+MNT	MNT
Vacuolization					
No vacuolization	0	8	0	2	8
Mild vacuolization (few isolated vacuoles present)	1	0	1	6	0
Moderate vacuolization (multiple vacuoles present in some areas)	2	0	5	0	0
Severe vacuolization (extensive vacuole formation throughout the nerve)	3	0	2	0	0
Edema					
No edema	0	8	0	2	8
Mild edema (intramyelinic)	1	0	4	6	0
Moderate edema (intraneuronal)	2	0	4	0	0
Severe edema (both intramyelinic and intraneuronal)	3	0	0	0	0
Infiltration					
No infiltration	0	8	0	4	8
Mild infiltration (few isolated infiltrating cells)	1	0	1	4	0
Moderate infiltration (multiple infiltrations in some areas)	2	0	6	0	0
Severe infiltration (widespread infiltration throughout the nerve)	3	0	1	0	0

MNT: montelukast; DTX: docetaxel.

**Table 3:** Statistical results of histopathological scores and immunohistochemically positive cell percentage of the groups.

	Control	DTX	DTX+MNT	MNT	P value
Vacuolization	0.00±0.00^a^	2.12±0.22^b^	0.50±0.18^a^	0.00±0.00^a^	<0.001
Edema	0.00±0.00^a^	1.50±0.18^b^	0.75±0.16^c^	0.00±0.00^a^	<0.001
Infiltration	0.00±0.00^a^	2.00±0.18^b^	0.50±0.18^a^	0.00±0.00^a^	<0.001
*Cas-3*	0.75±0.88^a^	18.50±0.70^b^	9.75±0.45^c^	0.74±0.26^a^	<0.001
*Bax*	0.62±0.26^a^	9.62±0.53^b^	2.87±0.29^c^	0.25±0.16^a^	<0.001
*Bcl-2*	33.12±0.97^a^	1.75±0.59^b^	12.25±0.64^c^	38.25±0.64^d^	<0.001

Results are reported as means±SE. The relationships between groups and histopathological and immunohistochemical results were compared by one-way ANOVA. *Post hoc* Dunnett C test was used for histopathological scores and *post hoc* Tukey for immunohistochemically positive cell numbers. Means of groups with different letters are significantly different. DTX: docetaxel; MNT: montelukast; *Cas-3:* caspase-3; *Bax: Bcl-2*-associated × protein; *Bcl-2:* B-cell lymphoma gene-2.

**Figure 8:**
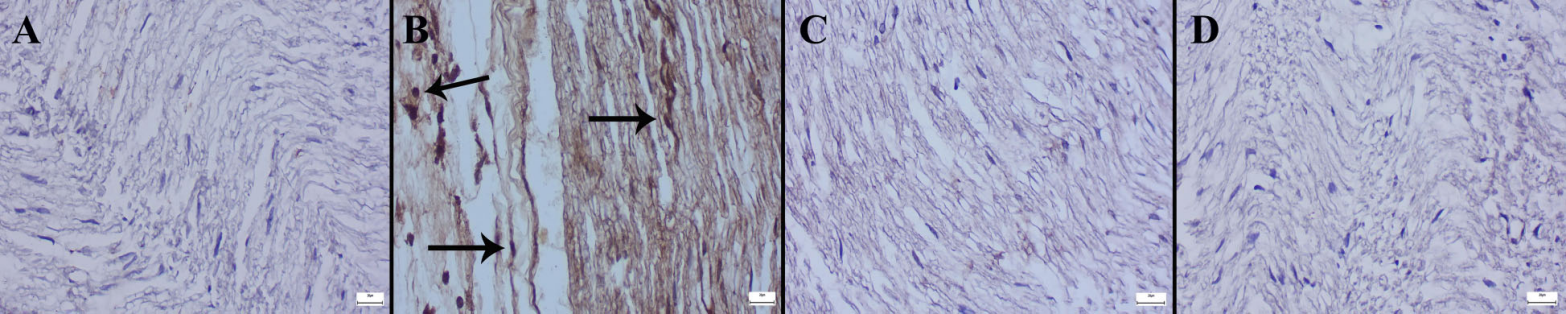
Caspase-3 immunohistochemical findings in the sciatic nerve (streptavidin-biotin peroxidase). **A**, Negative expression in the Control group. **B**, Marked increase in expression (arrows) in the DTX group. **C**, Decreased expression in the DTX+MNT group. **D**, No expression in the MNT group. Scale bars=20 µm. MNT: montelukast; DTX: docetaxel.

Examination of *Cas-3*, *Bax*, and *Bcl-2* immunostained preparations revealed that while DTX increased *Cas-3* and *Bax* expressions, it caused significant decreases in *Bcl-2* expressions. MNT treatment ameliorated the immunohistochemical scores (Figures 8-10). The statistical analysis results of the immunohistochemical scores of groups are shown in Table 3.

**Figure 9 f09:**
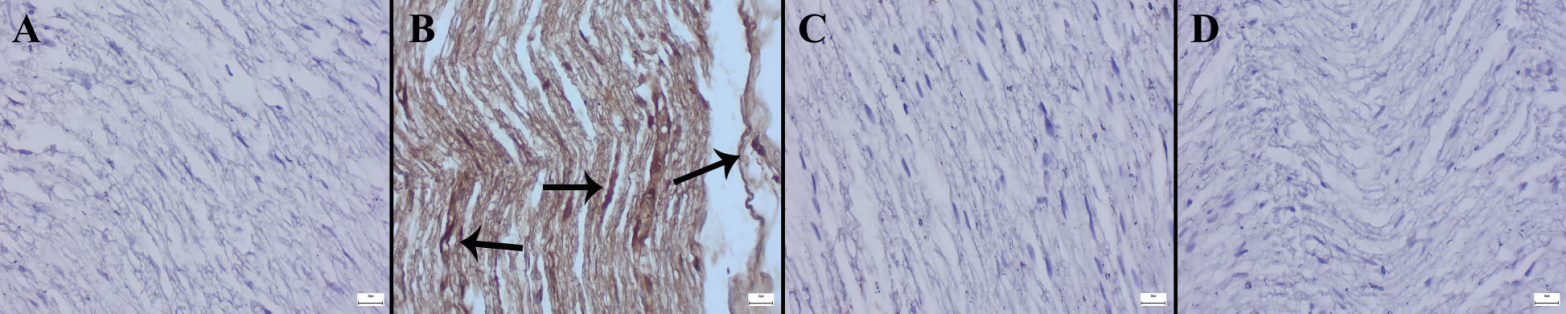
Representative images of *Bax* immunohistochemical findings in sciatic nerve between the groups (streptavidin-biotin peroxidase). **A**, No expression in the Control group. **B**, Marked increase in expression (arrows) in the DTX group. **C**, Decreased expression in the DTX+MNT group. **D**, Negative expression in the MNT group. Scale bars=20 µm. MNT: montelukast; DTX: docetaxel.

**Figure 10:**
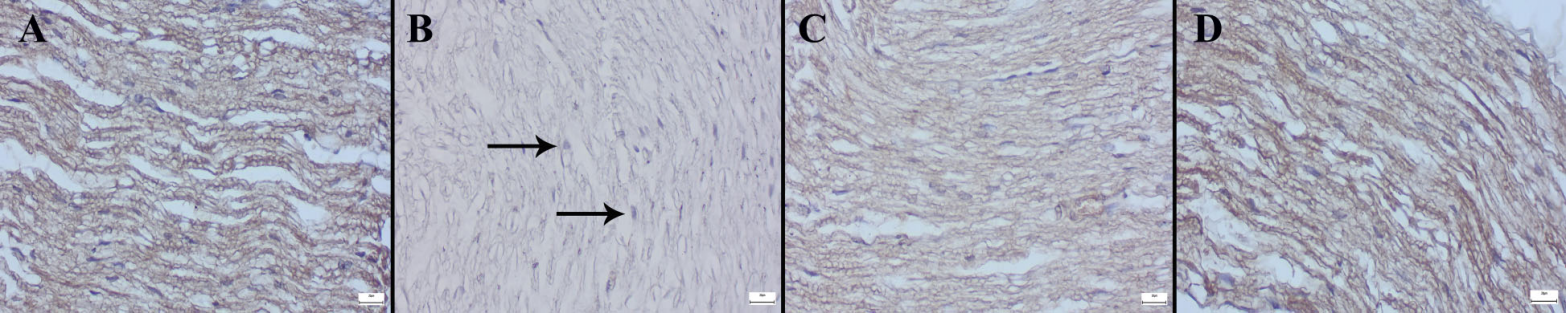
*Bcl-2* immunohistochemical findings of sciatic nerve among the groups (streptavidin-biotin peroxidase). **A**, Marked expression in the Control group. **B**, Markedly decreased expression (arrows) in the DTX group. **C**, Increased expression in the DTX+MNT group. **D**, Marked expression in MNT group. Scale bars=20 µm. MNT: montelukast; DTX: docetaxel.

The findings of this study demonstrated that DTX caused PN and that MNT had ameliorative effects against DTX-induced neuronal damage.

## Discussion

MNT, a medication used for over 20 years to treat asthma, allergic rhinitis, and urticaria, has recently been reported to exhibit neuroprotective activity in addition to its antioxidant and anti-inflammatory properties (10-12). These properties suggest that MNT has potential benefits in alleviating DTX-induced neurotoxicity. However, it has been reported that the long-term use of MNT may be associated with uncommon, dose-dependent neuropsychiatric events such as anxiety, depression, and, in rare cases, suicidal ideation (24). Nerve cells are particularly vulnerable to oxidative stress due to their high oxygen consumption and the abundance of polyunsaturated fatty acids (25). DTX induces excessive reactive oxygen species (ROS) production, leading to mitochondrial dysfunction, neurodegeneration, and neuronal apoptosis (6). The significance of ROS-mediated oxidative stress in DTX-induced neurotoxicity has been highlighted in previous studies (13,25). SOD and CAT enzymes, as well as GSH, are endogenous antioxidant substances that play key roles in neutralizing ROS and protecting cells from oxidative stress (26). In our study, DTX-induced oxidative stress in sciatic nerve tissues resulted in a significant reduction in SOD and CAT activities and GSH levels alongside an increase in MDA levels, a marker of lipid peroxidation. These findings are consistent with previous studies examining the effects of DTX on oxidant and antioxidant parameters in the sciatic nerve (13,27). The antioxidant effects of MNT on sciatic nerve tissue have been reported in previous studies (28,29). In the current study, MNT administration led to a slight improvement in SOD and CAT activities and GSH levels while significantly reducing MDA levels. Our findings suggested that MNT improved antioxidant parameters and reduced lipid peroxidation in DTX-induced sciatic nerve toxicity.

NF-κB and p38α MAPK play key roles in inflammation and apoptosis, while NCAM does the same in neural development and synaptic plasticity (29,30). In conditions associated with inflammation, such as DTX-induced peripheral neurotoxicity, significant elevations in the levels of NF-κB and p38α MAPK were observed, accompanied by a decrease in NCAM levels. Conversely, MNT administration significantly reduced NF-κB and p38α MAPK levels in sciatic nerve tissues while markedly increasing NCAM levels. Moreover, when examining the serum levels of proinflammatory cytokines TNF-α and IL-6, we found a significant increase in both cytokines due to DTX. However, MNT treatment significantly reduced TNF-α and IL-6 levels. Our results aligned with previous studies using different toxicity inducers in rats that have demonstrated the neuroprotective and anti-inflammatory effects of MNT (28,29,31).

*Bcl-2* inhibits apoptosis by attaching to the outer mitochondrial membrane, while *Bax* promotes apoptosis by increasing the release of pro-apoptotic proteins, and *Cas-3* serves as the central executioner enzyme in cell death (32,33). In our study, DTX treatment increased *Bax* and *Cas-3* expression while reducing *Bcl-2* expression in sciatic nerve tissue, whereas MNT co-treatment reversed these effects and improved histopathological parameters such as edema, axonal vacuolization, and inflammation. Previous studies have reported that DTX increases *Bax* and *Cas-3* expression while reducing *Bcl-2* expression, whereas MNT exerts the opposite effect (33-36). In this context, the results of the current study aligned with the findings of previous research.

Significant improvements were observed in the motor performance and thermal pain sensitivity parameters that were impaired by DTX. However, the number of studies investigating the effects of MNT on these parameters in experimental animal models of neurotoxicity is rather limited. Our findings are consistent with a study that induced Parkinson's disease in rats and reported that MNT significantly increased the time to fall in the rotarod test compared to the control group (37). Conversely, another study indicated that MNT significantly decreased the thermal hyperalgesia threshold in the tail flick test compared to the control group (38). In our study, while no significant difference was observed between the MNT and control groups in the tail flick test, the MNT group demonstrated a significant improvement compared to the DTX group. Although the two cited studies utilized the same MNT dose, differences in drug administration routes and durations may account for the discrepancies in test outcomes.

The primary enzymes responsible for the biotransformation of MNT and DTX in humans are CYP2C8 and CYP3A4, respectively. CYP2C22 and CYP3A1 are rat orthologues of human enzymes (16-18). When a xenobiotic inhibits a phase I CYP450 enzyme, enzyme levels in the body do not immediately fluctuate. Although enzyme activity may temporarily decline, the body attempts to compensate for this loss, and over the long term, enzyme levels can increase through mechanisms involving the upregulation of CYP450 enzyme gene expression and synthesis (39). After a relatively long period, approximately one month, any differences in enzyme levels could provide insights into the potential inhibition or activation associated with MNT and/or DTX. To the best of our knowledge, the current study is the first to investigate the *in vivo* effects of the MNT and DTX combination on CYP450 enzyme levels. Our findings revealed no significant differences after one month of drug administration in the levels of CYP3A1 or CYP2C22 enzymes among the experimental groups. These results suggest that there were no significant alterations in enzyme expression or synthesis during this period.

Our study focused solely on changes in CYP3A and CYP2C enzyme levels among the groups and did not assess enzyme activity through the pharmacokinetic profile of a known substrate of these enzymes. In the research, we investigated the expression of specific genes involved in apoptosis. Conversely, we did not explore the expression of genes in other apoptosis-related pathways, such as the phosphatidylinositol 3-kinase (PI3K), protein kinase B (Akt), and mammalian target of rapamycin (mTOR) signaling pathways. These limitations should be noted in the context of our study.

In conclusion, the study demonstrated that oral treatment with MNT effectively ameliorated DTX-induced neurotoxicity in the sciatic nerve tissues. Our results confirmed the protective effects of MNT against DTX-induced neurotoxicity, as evidenced by molecular, biochemical, and histopathological findings. This protective effect is attributable to MNT's antioxidant activity and free radical scavenging properties. MNT is a promising therapeutic option for reducing DTX-induced peripheral neurotoxicity. Therefore, further studies are warranted to elucidate the mechanisms underlying this protective effect and to determine the optimal dose.
